# Relationship between Sprint Capacity and Acceleration of Wrists in Wheelchair Basketball Players: Design and Reliability of a New Protocol

**DOI:** 10.3390/ijerph181910380

**Published:** 2021-10-02

**Authors:** Amelia Ferro, Javier Pérez-Tejero, Guadalupe Garrido, Jorge Villacieros

**Affiliations:** 1 Department of Sports, Faculty of Physical Activity and Sport Sciences, Universidad Politécnica de Madrid, 28040 Madrid, Spain; 2 Department of Health and Human Performance, Faculty of Physical Activity and Sport Sciences, Universidad Politécnica de Madrid, 28040 Madrid, Spain; j.perez@upm.es (J.P.-T.); lupe.garrido.pastor@upm.es (G.G.); 3 Physical Education and Adapted Sports, Campus La Salle, 28023 Madrid, Spain; jvrodriguez@lasallesagradocorazon.es

**Keywords:** performance, kinematics, laser, computer vision, inertial device, IMU

## Abstract

The application of new technologies in wheelchair basketball (WB) is important for the advancement and improvement of athletic performance. The purposes of this study are twofold: (a) to develop a methodological design in order to assess WB players’ performance, using wireless inertial measurement units (WIMU^®^) and a laser system (BioLaserSport^®^ with computer vision), in a 20 m sprint test on court and (b) to assess bilateral symmetry as a performance indicator and for injury prevention purposes, the study of which in previous research is unknown. For both aims, the relation of the acceleration of the players’ wrists to the speed achieved by the player in the wheelchair was explored. Ten elite WB players participated in an on-court 20 m sprint test during real training. BioLaserSport^®^ with computer vision was used to assess the average velocity (Va) and maximum velocity (Vmax) of the WB players, and two WIMU^®^ were used for the total acceleration (AcelT) of the players’ wrists. A very high correlation was obtained in the assessment of the Va (0.97) and AcelT of both wrists (0.90 and 0.85). There was a significant relationship between the average AcelT of the dominant wrist and the Va on-court sprint velocity (*p* < 0.05). Two players did not show good wrist symmetry. In conclusion, a new methodological protocol was developed, making it possible to assess the bilateral symmetries in elite WB players in on-court real training and the relation between the acceleration of players’ wrists and players’ wheelchair speed. Coaches can use this protocol to assess performance or for injury prevention, as it shows very good reliability, with high ICC values.

## 1. Introduction

The application of new technologies in wheelchair basketball (WB) is important for the advancement and improvement of athletic performance. Initially, studies were carried out under laboratory conditions [1,2,3,4,5], but in recent years, real training conditions have been used [6,7,8,9].

In WB, the ability to sprint from a standstill condition is crucial. The sport-specific movement demands, established as starting, sprinting, braking, turning (pivoting) and blocking, are specifically related to the use of the wheelchair [10]. In addition, Mason et al. [11] have defined stability, initial acceleration, maneuverability and sprinting as critical areas for successful WB performance. Despite the importance of sprint capacity in this sport, it still has not been investigated in depth [3].

In the literature, several tests have been described for assessing sprint performance in WB players [7,9,12,13]; however, how the movements of the upper limbs can influence the propulsion cycle in gaining speed in the wheelchair requires further attention [14]. According to Rao et al. [15], the wrist participates very actively in the wheelchair propulsion cycle, from extension and radial deviation to flexion and ulnar deviation. For this reason, it is important to determine whether the acceleration produced by the wrist influences the increase in the players’ speed in the wheelchair, since it is the closest joint to the handrim.

Acceleration has been studied through inertial measurement systems (IMU), where its reliability and validity have been demonstrated [16,17,18]. This technology makes it possible to systematically monitor the movements of the human body when applied to competitive sports [19,20,21] and to obtain a 3D orientation of the body segments [16]. Bergamini et al. [6] presented a method to quantify the characteristics of WB players in a 20 m sprint test using IMU. Their results confirmed the importance of adequate propulsion symmetry as an indicator value related to athlete sport performance and potential injuries. It should be noted that this indicator has also been studied by other authors [22,23]. However, an accurate description of the protocol was not made for its measurement, and the use of a stopwatch to calculate the speed was not reliable [6]. In our opinion, it is necessary to develop an innovative methodology that combines force moments of the wrists along wheelchair propulsion, measured through isokinetic dynamometers, with sport-specific demands. In this regard, Wei et al. [24] explored wrist kinematics during wheelchair propulsion in a non-sportive population, finding seat height to be a factor influencing handrim contact time (the higher the seat, the lower the handrim contact time, wrist extension angles and range of motion). However, no studies on wheeled sport, such as WB, were found. Furthermore, to our knowledge, no study has taken into account the acceleration generated by the wrist joints in WB-specific environments. This joint is the closest to the propulsion ring in the wheelchair, so it is probably the one that suffers the most impact [24], especially when performing a sprint. For this reason, it is necessary to design a protocol that places measurement systems on the wrists in order to relate the acceleration of these joints to the speed achieved by the player during the sprint and detect possible bilateral symmetries that may occur during the sprint. This should also be analyzed taking into account functional classification, as it is a factor influencing players’ wheelchair configuration and seating height [10,24].

This gap in the previous literature should be addressed by a combination of technological developments that bring innovative options for WB players and coaches, as sprinting from a stand-still position is considered a key performance ability in this sport. For all of the above, and in the authors’ opinion, the reliability of the published protocols to analyze sprint capacity in WB players is still limited. The purposes of this study are (a) to develop a reliable methodological design in order to assess sprint performance of WB players, using IMU and a laser system with computer vision in a 20 m sprint test, and (b) to assess bilateral symmetry as an indicator of sport performance and as an injury prevention indicator in WB players. Thus, the relation of the total acceleration of the players’ wrists to the speed achieved by the WB players was explored during an on-court 20 m sprint test as an indicator of improvement in performance.

## 2. Materials and Methods

### 2.1. Participants

Ten elite WB players (8 men and 2 women) participated in the study. Throughout the study period, these players won first positions in the National League, the National King’s Cup and the European Cup for clubs. In addition, seven of them were medalists in the Rio de Janeiro Paralympic games. All were classified by the International Wheelchair Basketball Federation (IWBF) Player Classification System [25] (Table 1). Their height was measured using a stadiometer in a standing situation (DKSH Switzerland Ltd., Zurich, Switzerland, ±0.1 cm) Body weight was assessed using a calibrated scale (Kern MWS, Twister Medical, Barcelona, Spain, ±0.1 kg). Players were first weighed in their own sport wheelchairs; then, the wheelchair was weighed without them, and the wheelchair weight was then calculated.

All of the players trained 4–5 days per week, playing one game per week. The study was undertaken during the competitive period. During data collection, no participants experienced injuries that could have potentially influenced their ability to perform training or research tasks. All participants were selected during the preparation–screening period. The study was approved by the University Ethics Committee, and it was undertaken according to the Helsinki Declaration on research in humans [26]. Information about the study aims was provided, and informed consent was obtained prior to the study and data collection. All of the subjects wore sports gear specific to the sport and used their sports wheelchair. In addition, the strapping and wheelchair configuration were chosen by every player for training and competition, as the players’ functional classifications are valid in these circumstances [2].

### 2.2. Equipment

Distances were measured using a laser sensor-type 1 LDM301 (Jenoptik, Jena, Germany) with a range of 0.5–300 m on natural surfaces, an accuracy of ±0.06 m for 2 kHz and a resolution of 0.001 m. In addition, a laser TLM160i (Stanley, Mechelen, Belgium), calibrated according to the ISO standard, was used to measure test distances. Labview software, v. 13.0 (National Instruments, Austin, TX, USA), was used for data recording and processing to obtain velocities. All of the components were integrated into a BioLaserSport^®^ (Ferro, A., Madrid, Spain) [27], real-time kinematic analysis system for training and sports competitions (UPM-UPO, Madrid, Spain) [28], which included a computer vision system developed by visual algorithm tracking in C++ [29], and which validity and reliability were calculated in previous studies using a 30 m sprint test [30]. High correlation coefficients of 0.962 for mean velocity (Va) were found with regard to the photocell systems and 0.869 for maximum velocity (Vmax) with regard to high-speed photogrammetry. For intrasession reliability, intraclass correlation coefficients (ICC) showed values of 0.945 for Va and 0.866 for Vmax, with 95% confidence intervals (CI) [30].

Two multi-sensor wireless inertial measurement units (WIMU^®^ v. 1.6., Real Track System S.L, Almería, Spain) were used. These devices contained two three-axial accelerometers (400 g of full range scale and recording at 1000 Hz), which provided the components of the vector sum of gravitational and inertial linear accelerations. The data were processed with Qüiko software v.882 (Realtrack Systems, S.L. Almería, Spain).

### 2.3. Design and Procedures

**Figure 1:**
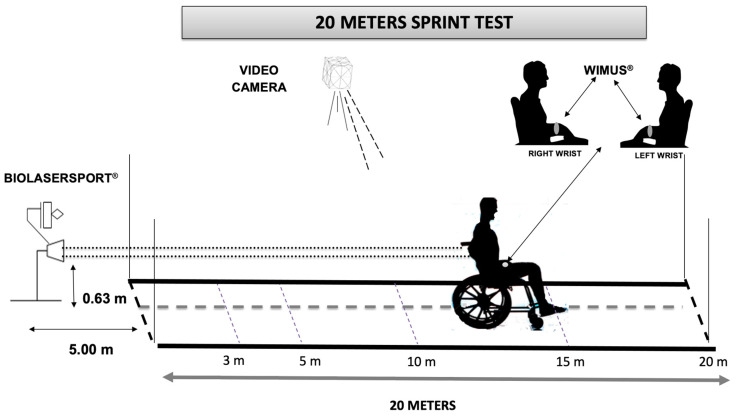
Diagram of field 20 m sprint test.

The 20 m sprint test (Figure 1) was performed according to [7], in a real wooden facility with a training basketball court. Players were asked to inflate their tubes up to the maximum, as during a competition. The test started with a standard 15 min warm-up directed by the coach, which included continuous wheeling, joint mobility, and stretching of the upper limbs in static and dynamic situations. The athletes were positioned at the start line with the front wheelchairs’ castors over the line and the players’ trunk behind the same line. Two posts were placed at the end of each line, to ensure that the players’ chests or the wheels did not cross before the start of the test. Starting verbal signals used were “ready” and then “when you like”, so the players were free to perform preparatory driving movements with their trunks and to start propelling the wheelchair forward when they were ready, avoiding the undesirable effects of the players’ reaction times on sprint performance. The tests were performed twice, and the end score was the mean of the two trials. The laser was situated 5 m from the starting line and the laser beam hit the players’ backs at a height of 0.63 m from the ground, while the laser beam was controlled its horizontallity A 2000 Hz sampling frequency was selected for data position recording. Data were then filtered at 3 Hz with a second-order Butterworth low-pass filter. Then, maximum velocity (Vmax) and average velocity (Va) were calculated in the sections: 0–3, 3–5, 5–10, 10–15 and 15–20 m.

Both WIMU^®^ were fastened on the players’ right and left wrists using elastic bands. Players were in an upright position while devices were placed. The ulnar and radial styloid apophysis were located and, from that point on, the entire forearm was supported. This allowed the wrist total freedom of movement during the propulsion of the wheelchair. To remove random noise, the optimal cutoff frequency was established using a low-pass filter for speeds greater than 1.8 m/s [31]. In our case, the measured accelerations were low-pass-filtered with a cut-off frequency of 12 Hz using a 4th-order zero-lag Butterworth filter [6]. Accelerometer calibration was verified and checked at the beginning of the experimental session. Two video cameras (Exilim EX-ZR1000, Tokio, Japan) were placed on each side of the 20 m sprint test. All attempts were recorded at 240 Hz, and a synchronism signal was sent from 2 WIMU^®^ and recorded to detect the propulsion cycles of each WB player. Based on the team’s technical staffs’ advice, for each session/player/trial, the total acceleration in each propulsion cycle (AcelTp) and the average and maximum AcelT were recorded in the indicated five different sections. Bilateral symmetry between the dominant and non-dominant wrist was computed from AcelT in each propulsion cycle according to Bergamini et al. [6]. The calculation was carried out using the following algorithm *sym = [AcelTp dominant/(AcelTp dominant + AcelTp non-dominant)] × 100*. To study if the dominant and non-dominant wrist presented similar peak accelerations [6], bilateral symmetry with values ranging between 45% and 55% indicated adequate symmetry, whereas a value lower than 45% or higher than 55% reflected greater accelerations of the non-dominant or dominant wrist, respectively [23].

### 2.4. Statistical Analysis

Descriptive analyses were carried out for each test. The Shapiro–Wilk test was used to assess the normality of the tested variables. For intrasession reliability, the relationship between the results of AcelTp in the first and the second test series was analyzed using the ICC (2,1) as well as a CI of 95%. CI estimation provides a range of values with a specific probability, including true reliability [32]. The standard error of measurement (SEM) was calculated as $\left(\mathrm{SD}\right)\sqrt{1.00-\mathrm{ICC}}$, where the standard deviation (SD) was the deviation of the observed scores. The relationships between the average and maximum AcelT, Va and Vmax were examined using Pearson’s product–moment correlation coefficient (r). The significance level was determined at *p* < 0.05. All of the calculations were performed with the *SPSS* software program, version 21.0 (IBM Corp., Armonk, NY, USA).

## 3. Results

### 3.1. Twenty-Meter Sprint Test Protocol Reliability Using Two WIMU

Table 2 and Table 3 show the average AcelTp (in each of the nine propulsion cycles) obtained for all the players in the 20 m sprint test, as the data allowing intrasession reliability assessment indicated no significant differences between the different series. Average ICC values were 0.90 for WIMU-1 on the non-dominant side and 0.85 for WIMU-2 on the dominant side, both with 95% CI and an average SEM of 1.15 and 1.24 g, respectively.

### 3.2. Twenty-Meter Sprint Test Protocol Reliability Using BioLaserSport^®^ with Computer Vision System

Table 4 presents the intrasession reliability from the average Vmax assessment obtained during the 20 m sprint test for all the players, not observing significant differences between each series. Average ICC values were 0.967 for all Vmax sections with 95% CI and SEM values of <0.05 m/s.

### 3.3. Players’ Wrist Bilateral Symmetry in Two Series of 20 M Sprint Tests

**Figure 2:**
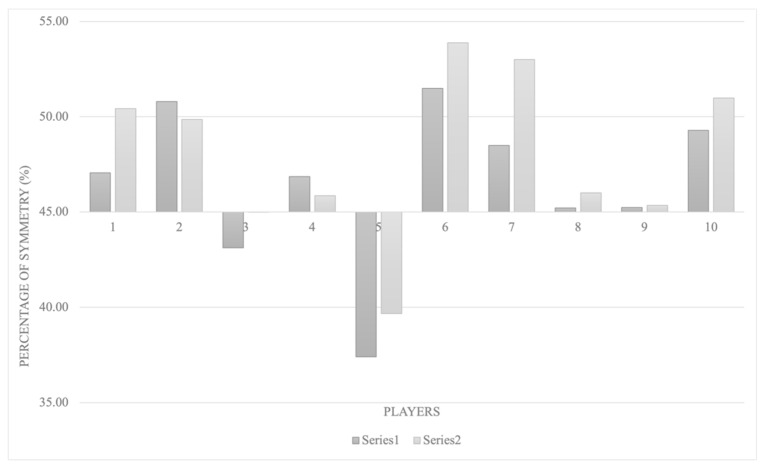
Bilateral symmetry for all the WB players in two series of 20 m sprint tests.

Figure 2 shows the symmetry between dominant and non-dominant wrists during the tests. Two players did not present adequate bilateral symmetry in the two 20 m sprint tests because they were not between the 45–55% range of values. The remaining players showed adequate symmetry.

### 3.4. Relation between Average/Maximum Total Acceleration and Average/Maximum Velocity from Dominant/Non-Dominant Players’ Wrists

**Figure 3:**
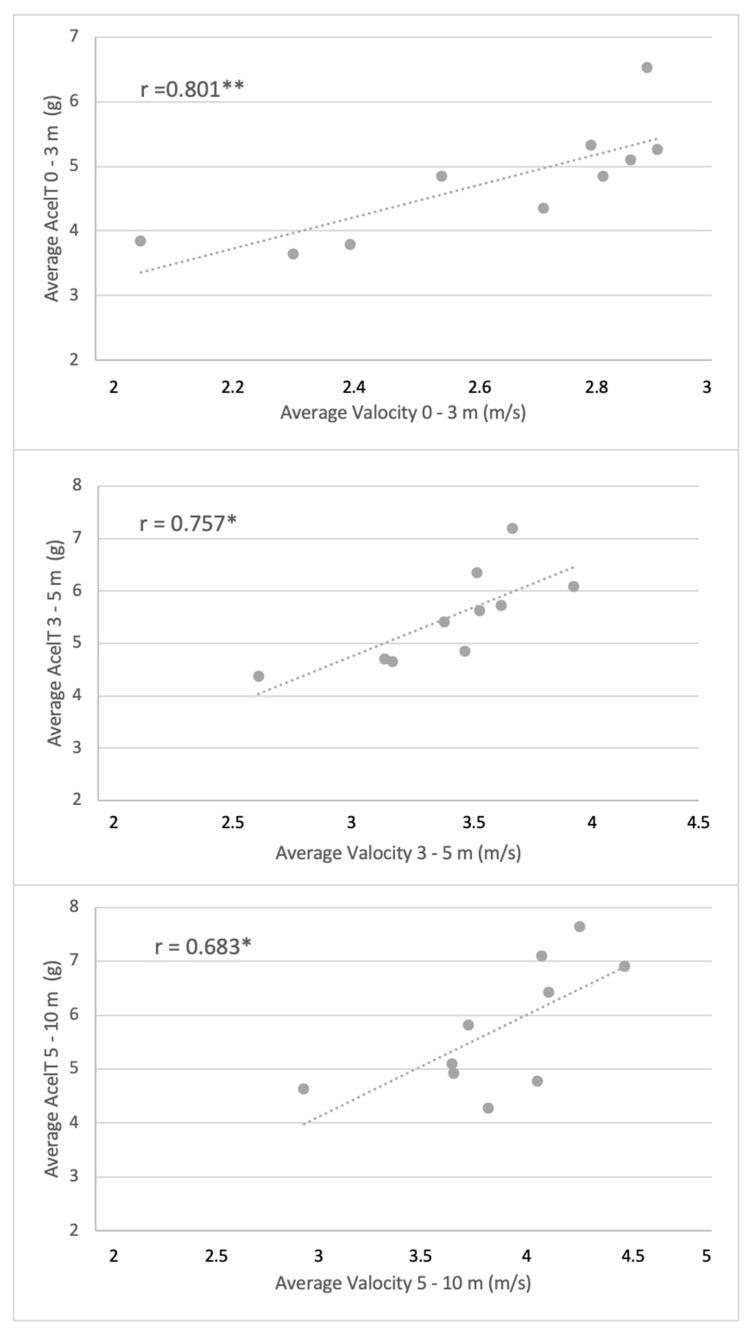
Correlations between the average velocity (Va) and the average total acceleration (AcelT) of the dominant side in the 20 m sprint test sections: 0–3 m, 3–5 m and 5–10 m (* *p* <0.05, ** *p* <0.01).

Significant correlations were found for the players’ dominant wrists in 0–3 m, 3–5 m, and 5–10 m between the average velocity and average AcelT (Figure 3) (*p* < 0.05). However, for the maximum AcelT and Vmax values, no significant correlations were found, with none found for the non-dominant side.

## 4. Discussion

This original study presents a new and reliable methodology for WB players’ sprint assessments in real sport training conditions using two new technologies, WIMU^®^ and BioLaserSport^®^ with a computer vision system (inertial devices and laser system). The analysis of the players’ sprint capacity in relation to wrist acceleration seems important, as sprinting is a crucial skill in WB [33], with remarkable and novel significance for players and coaches. With objective data gathering from wrist acceleration using WIMU, sprinting was found in our study to be a key component in overall wheeling abilities and is also known for its potential injury incidence in this population [34]. Moreover, our analysis was conducted in a sport context, so application and transfer of these findings to a specific training assessment and screening is possible, especially with an elite-level sample. To our knowledge, this study is the first to relate the wrist triaxial acceleration of WB players to their speed achieved in an on-court 20 m sprint test.

First, there was a need to verify protocol reliability for the 20 m sprint test using the BioLaserSport^®^ and the WIMU^®^ systems. Using computer vision, we obtained an average ICC of 0.967 in Vmax and a lower SEM of 0.05 m/s. Comparing our tests with those performed by Ferro et al. [7], the use of the laser system with computer vision further improved the reliability of the calculation of the WB players’ velocity by 7%. In relation to the use of WIMU^®^, following Bergamini et al. [6], this proposed protocol is not clear. The placement of their WIMU^®^ on the wrists in that study was not well specified, so study reproducibility is not possible, and they do not provide data on measurement reliability. In our study, WIMU^®^ placement was described, and reliability data were presented, showing an ICC of 0.90 and 0.85 for players’ non-dominant and dominant wrists, and a SEM between 0.79 and 1.71 g was recorded. As a consequence, the acceleration data in our study were reliable. The combination of these two technologies provides this study with innovation and significance for future practical applications in real sport-specific WB contexts.

It is important that the technologies used in the analysis of athletic performance have certain criteria and rigor, as is the case of the WIMU^®^ and the BioLaserSport^®^ with computer vision used in this research. Some studies that analyze the performance velocity of athletes using a stopwatch have a limitation on the application of the results because of the less reliable system [6,12,13,35]. Similarly, and in order to assess sprint capacity in WB players, some previous studies [7,8,13,36] have used a speed test, with the 20 m sprint being the most common. However, no studies have been found in the literature observing the importance of wrist action with the speed achieved by the WB player. In our study, a significant correlation was observed between the average velocity of the WB players with an average acceleration of the dominant wrist of the players in the 0–3 m (r = 0.801), 3–5 m (r = 0.757) and 5–10 m sections (r = 0.683) (Figure 3). We observed that this relationship is stronger at the beginning of the test (in the 0–3 m section versus the 5–10 m section). We could conclude that the acceleration produced by the dominant wrist is more crucial at the start of the sprint. We found studies that have analyzed the speed in the first few meters of a sprint [7,37], which suggests the importance of this ability at the start of a sprint, to enable the player to achieve very early speeds in order to intercept the opponent’s trajectory during the actions of a basketball game [10].

Regarding the second aim dealing with bilateral symmetry assessment, we found articles that analyzed it but failed to identify the differences between the dominant and non-dominant sides [6,12]. In our case, two participants did show differences between the sides (Figure 2), as they showed a relationship below 45% [23]. These findings were not associated with the players’ functional classification; this finding appears to be in line with the conclusions of Wai et al. [24], as no differences were found in the wrist accelerations and seating height during manual wheelchair propulsion within a non-sportive population. In this study, the differences in one of the cases were very small; however, the high precision of the devices used and the reliability of the protocol could detect them. Again, we emphasize the importance of using reliable protocols to evaluate performance in high-level athletes.

This study was carried out with 10 athletes. This could be initially considered a limitation. However, the data could be used as a reference because of the participation of high-level athletes. They achieved first place in three competitions in one season, the National League, the European Cup and the National King’s Cup, and, in addition, seven of the participants were Paralympic medalists. For this reason, the results of the study are especially important. In addition, two female players participated in the study; in most clubs at national level, women participate together with men in competitive WB in order to promote their participation in this sport. If the coach allows one female player to play on court, the sum of the functional classification of the five players on court can be up to 16 points (plus 1.5 points), instead of 14.5 points when only males are on court. In our opinion, their presence gives contextual validity to the results, as we know the two female players have also reached the Paralympic level in their careers. 

In future investigations, it would be interesting to carry out studies where the joint patterns during the propulsion cycle are analyzed in more detail, as well as identifying how they influence speed gains during WB sprint performance. We should consider optimizing mobility performance in court sports (wheelchair basketball, wheelchair rugby and wheelchair tennis), as this depends on an ergonomic combination of factors associated with the player, their own sport wheelchair and the interface between them [10,38].

## 5. Conclusions

In conclusion, this study presents a new, reliable and valid protocol, using WIMU^®^ and BiolaserSport^®^ with computer vision, to assess bilateral symmetries during sprint performance in WB players. This protocol demonstrated the relationship between the players’ velocity of movement and the generated acceleration of the players’ wrists. It was observed that there was a significant relationship between the average acceleration of the dominant wrist and the average velocity of the player in the wheelchair, with this relationship being stronger at the beginning (0–3 m) of the 20 m sprint test. Coaches can use this protocol to assess performance or for injury prevention, as it showed very good reliability, with very high ICC values.

## Figures and Tables

**Table 1:** Main characteristics of the wheelchair basketball players in the study.

Player	Age	Weight (Kg)	Height (m)	Disability	IWFB Classification	Experience (Years)
P1	33	74	1.80	Amputation	4.5	20
P2	39	75	1.86	Amputation	4.0	22
P3	28	75	1.86	Amputation	4.0	8
P4	39	90	1.82	Paraplegia	4.0	21
P5	17	60	1.84	Amputation	3.5	5
P6	40	90	1.82	Spina Bifida	3	10
P7	26	83	1.78	Spina Bifida	3	8
P8	23	72	1.75	Paraplegia	2.5	6
P9	30	55	1.50	Paraplegia	1	18
P10	33	102	1.80	Paraplegia	1	17
Sample	30.80 ± 7.54	77.60 ± 14.18	1.78 ± 0.11			13.50 ± 6.70

**Table 2:** Intrasession reliability for the average total acceleration in each of the nine propulsion cycles of the non-dominant wrists of all the players during two series of 20 m sprint tests (using WIMU-1).

Variable/ Cycle 1–9	Test 1	Test 2	ICC	SEM
			(95% IC)	(g)
AcelTp 1 (g)	10.03 ± 2.02	9.69 ± 1.87	0.796 (0.393–0.944)	0.84
AcelTp 2 (g)	12.10 ± 2.52	11.89 ± 2.65	0.770 (0.304–0.938)	1.20
AcelTp 3 (g)	13.15 ± 3.48	12.64 ± 3.13	0.854 (0.432–0.950)	1.20
AcelTp 4 (g)	14.12 ± 3.98	13.96 ± 4.26	0.959 (0.846–0.990)	0.81
AcelTp 5 (g)	14.76 ± 4.29	14.81 ± 5.10	0.961 (0.850–0.990)	0.85
AcelTp 6 (g)	15.70 ± 5.15	15.04 ± 5.83	0.962 (0.858–0.990)	1.00
AcelTp 7 (g)	16.18 ± 5.22	15.87 ± 5.68	0.944 (0.796–0.986)	1.24
AcelTp 8 (g)	17.42 ± 6.10	16.20 ± 5.99	0.924 (0.709–0.981)	1.65
AcelTp 9 (g)	17.94 ± 6.37	16.30 ± 6.09	0.930 (0.483–0.985)	1.61

AcelTp: total acceleration in each propulsion cycle. ICC: intraclass correlation coefficient. CI: confidence interval. SEM: standard error of measurement.

**Table 3:** Intrasession reliability for the average total acceleration in each of the nine propulsion cycles of the dominant wrists of all the players during two series of 20 m sprint tests (using WIMU-2).

Variable/ Cycle 1–9	Test 1	Test 2	ICC	SEM
			(95% IC)	(g)
AcelTp 1 (g)	9.22 ± 1.65	9.49 ± 1.95	0.772 (0.327–0.938)	0.79
AcelTp 2 (g)	10.83 ± 2.19	10.44 ± 1.88	0.772 (0.304–0.937)	0.90
AcelTp 3 (g)	11.62 ± 3.10	11.83 ± 3.02	0.855 (0.519–0.962)	1.15
AcelTp 4 (g)	12.39 ± 3.18	12.40 ± 3.08	0.869 (0.446–0.968)	1.11
AcelTp 5 (g)	13.69 ± 4.27	13.30 ± 3.63	0.866 (0.371–0.970)	1.33
AcelTp 6 (g)	13.45 ± 4.34	13.36 ± 3.76	0.884 (0.516–0.972)	1.28
AcelTp 7 (g)	14.39 ± 4.96	13.90 ± 3.84	0.855 (0.410–0.964)	1.46
AcelTp 8 (g)	14.76 ± 5.16	14.19 ± 4.61	0.863 (0.446–0.966)	1.71
AcelTp 9 (g)	14.47 ± 4.65	14.68 ± 4.76	0.904 (0.602–0.976)	1.44

AcelTp: total acceleration in each propulsion cycle. ICC: intraclass correlation coefficient. CI: confidence interval. SEM: standard error of measurement.

**Table 4:** Intrasession reliability for average maximum velocity for all the players during two series of 20 m sprint tests.

Variable/ section	Test 1	Test 2	ICC	SEM
			(95% IC)	(m/s)
Vmax 0–3 (m/s)	3.41 ± 0.27	3.44 ± 0.28	0.962 (0.851–0.981)	0.05
Vmax 3–5 (m/s)	3.97 ± 0.21	4.01 ± 0.16	0.962 (0.862–0.990)	0.04
Vmax 5–10 (m/s)	4.65 ± 0.22	4.75 ± 0.29	0.958 (0.851–0.989)	0.05
Vmax 10–15 (m/s)	5.05 ± 0.26	5.09 ± 0.32	0.976 (0.913–0.994)	0.05
Vmax 15–20 (m/s)	5.30 ± 0.29	5.33 ± 0.29	0.970 (0.885–0.992)	0.05

Vmax: maximum velocity. ICC: intraclass correlation coefficient. CI: confidence interval. SEM: standard error of measurement.

## Data Availability

Not applicable.
